# The defining features of intrinsic transcription terminators

**DOI:** 10.64898/2026.05.29.728758

**Published:** 2026-05-31

**Authors:** Robert A. Battaglia, Ebru Ermiş, Expery O. Omollo, Gene-Wei Li

## Abstract

Transcription terminators are universal landmarks that delimit RNAs and tune downstream gene expression, yet the sequence rules that define them remain elusive. The classical model of bacterial intrinsic termination—an RNA hairpin followed by a uracil-rich tract—is incomplete, as stem-preserving mutations can abolish termination. By precisely mapping termination sites across 10 ^4^ sequences, we uncovered a previously unresolved feature required for intrinsic termination: dinucleotides positioned at both edges of the transcription bubble, resembling the elemental RNA polymerase pause signal. Together, hairpin, U-tract and bubble-edge sequences (HUB) account for most variation in termination efficiency and pinpoint bona fide terminators across diverse bacterial phyla. These findings establish HUB as the defining element of intrinsic terminators and provide a framework for decoding and engineering gene expression across genomes.

## Full Text Availability

The license terms selected by the author(s) for this preprint version do not permit archiving in PMC. The full text is available from the preprint server.

